# Sex-specific metabolic effects of Treg expansion in db/db mice

**DOI:** 10.3389/fimmu.2025.1599985

**Published:** 2025-10-27

**Authors:** Daniel Pérez-Martínez, María Jose Canz, Elisabet Torras-Peyrotón, Anna Alvarez-Guaita, Lorena Ramos-Pérez, Cristina Hernández, Rafael Simó, María Llorián-Salvador

**Affiliations:** ^1^ Diabetes and Metabolism Research Unit, Vall d’Hebron Research Institute (VHIR), Barcelona, Spain; ^2^ Centro de Investigación Biomédica en Red de Diabetes y Enfermedades Metabólicas Asociadas (CIBERDEM), Instituto de Salud Carlos III, Madrid, Spain; ^3^ Universitat Autònoma de Barcelona, Barcelona, Spain

**Keywords:** sex differences, regulatory T cells, immunomodulation, inflammation, type 2 diabetes, metabolism

## Abstract

**Introduction:**

Type 2 diabetes mellitus (T2D) is characterized by chronic, low-grade inflammation and immune dysregulation, contributing to insulin resistance and metabolic complications. Regulatory T cells (Tregs) are key modulators of immune homeostasis, and their therapeutic expansion has shown promise in limiting inflammation. Here, we investigated whether *in vivo* Treg expansion could modulate metabolic and inflammatory responses in T2D in a sex-specific manner.

**Methods:**

Male and female db/db and db/+ mice received intraperitoneal injections of IL-2/anti-IL-2 (JES6-1) complexes for six weeks to induce endogenous Treg expansion. Body weight, blood glucose, and insulin levels were monitored to calculate HOMA-IR. Plasma inflammatory and anti-inflammatory mediators (CRP, adiponectin, IL-10, IL-13, CCL3) were quantified by bead-based immunoassays. Hepatic triglycerides and gene expression of Foxp3, F4/80, Pparγ, Il10 and Il13 were analyzed by biochemical assays, western blot, and RT-qPCR.

**Results:**

Treg expansion reduced weight gain in db/db mice independently of food intake. Female mice displayed lower HOMA-IR, decreased CRP, and increased adiponectin, IL-10, and IL-13 levels, whereas males showed limited improvement. Hepatic triglycerides and F4/80 expression were reduced after Treg expansion, with restored Foxp3 and elevated Il10/Il13 expression, indicating diminished hepatic inflammation.

**Conclusion:**

Endogenous Treg expansion exerts sex-specific metabolic and anti-inflammatory benefits in db/db T2D mice, with females showing greater improvements in insulin resistance, inflammation, and hepatic lipid metabolism. These findings support Treg-based immunomodulation as a potential therapeutic approach for T2D and emphasize the need for sex-specific considerations in future research.

## 1 Introduction

Type 2 diabetes mellitus (T2D) has become a prominent global health challenge, with recent estimates indicating that approximately 537 million adults were living with diabetes worldwide in 2021, a number projected to reach 783 million by 2045 (1). Accounting for over 90% of all diabetes cases, T2D imposes a substantial burden on healthcare systems and significantly reduces patient quality of life, primarily through its associated microvascular and macrovascular complications (2).

A hallmark of T2D is chronic, low-grade inflammation, primarily driven by adipose tissue dysfunction (3, 4). Contributing mechanisms include hypoxia and adipocyte cell death, activation of the nuclear factor-κB (NF-κB) and JUN N-terminal kinase (JNK) pathways, interleukin-1β (IL-1β) signaling, and immune cell recruitment. Increasing evidence highlights the critical interplay between innate and adaptive immune dysregulation in the development and progression of T2D (3–7).

T2D is characterized by significant immune dysfunction, including impaired macrophage, neutrophil, and natural killer (NK) cell activity, which increases susceptibility to infections, systemic inflammation, and disease complications. In insulin-resistant tissues, macrophages shift to a pro-inflammatory state, producing elevated levels of cytokines and chemokines that sustain chronic inflammation (3, 4, 7). Additionally, heightened neutrophil activation and inflammasome activity further increase the inflammatory responses. Dysfunctional antigen-presenting cells, including dendritic cells, contribute to immune dysregulation, while altered NK cell function exacerbates inflammation and metabolic imbalance (3, 4).

The adaptive immune system undergoes significant alterations in T2D, contributing to chronic inflammation and insulin resistance. Activated T lymphocytes, particularly CD8+ T cells, accumulate in adipose tissue, where they exacerbate inflammation and insulin resistance by releasing interferon-gamma (IFN-γ), tumor necrosis factor-alpha (TNF-α), and other pro-inflammatory mediators, thereby amplifying local inflammatory circuits. Similarly, B lymphocytes play a pathogenic role by producing autoantibodies and modulating T cell and macrophage responses (4). Specifically, B cells promote insulin resistance and glucose intolerance by activating Th1 and Th17 cells and secreting pro-inflammatory antibodies.

CD4^+^ T cells further contribute to metabolic dysfunction by shifting the immune balance toward a pro-inflammatory state. Th1 and Th17 cells are elevated in peripheral blood and adipose tissue of T2D patients, whereas Th2 and regulatory T cells (Tregs) are reduced or present in an imbalanced state (4, 8, 9), thus exacerbating insulin resistance and systemic inflammation. Overall, this maladaptive immune response perpetuates chronic inflammation, increases the insulin resistance, and accelerates T2D-associated complications.

Tregs play a critical role in immune regulation. A systematic review and meta-analysis reported a significant reduction in peripheral CD4^+^Foxp3^+^ Tregs in patients living with T2D compared to healthy controls, with even greater reductions observed in those with diabetes-related complications (9). However, discrepancies exist in literature. While some studies do not report a reduction in Treg numbers, they highlight an imbalance between Th17 and Treg cells (8), whereas others suggest a decline in Treg functionality (10).

Despite these variations, accumulating evidence suggests that altered Treg homeostasis—whether through numerical depletion or impaired function—contributes to the chronic inflammatory environment in T2D. We have recently reported a reduced percentage of CD4^+^CD25^+^Foxp3^+^ Tregs in the blood of db/db mice compared to non-diabetic controls, a finding particularly relevant given the strong similarity of db/db mouse model to human T2D (11). Supporting this, Eller et al. (12) demonstrated that Treg depletion in db/db mice exacerbates insulin resistance and nephropathy, whereas Treg administration improves insulin sensitivity and mitigates diabetic nephropathy. These findings highlight the potential of Tregs as therapeutic targets in modulating inflammation and metabolic dysfunction in T2D. Tregs are modulated by immunometabolic signals, with leptin acting as a key negative regulator via its receptor (ObR), which is highly expressed on Tregs. Disruption of this pathway—through leptin neutralization or deficiency—has been shown to enhance Treg proliferation and function (13, 14). Although not directly examined in this study, the leptin–Treg axis is particularly relevant in the leptin receptor–deficient db/db mouse model, which closely mirrors human T2D. Our findings, alongside prior reports, highlight the complex and context-dependent nature of leptin’s effects on Tregs—shaped by factors such as disease stage, tissue environment, and metabolic status—potentially explaining discrepancies in Treg responses across studies and supporting the rationale for investigating Treg expansion in this model.

Changes in immune-response is an important pathogenic factor in T2D (3–7). In addition, females generally exhibit stronger innate and adaptive immune responses than males (15). On this basis, the main aim of the present study is to examine the impact of Treg Expansion on body weight, insulin resistance metabolic and inflammation parameters in db/db mice.

## 2 Methods

### 2.1 Animals

Diabetic male db/db (BKS.Cg-Dock7m +/+ Leprdb/J) mice and their control, non-diabetic mice (db/+; (BKS.Cg-Dock7m + Leprdb/+) were acquired from Charles River Laboratories (Calco, Italy) at 6 weeks of age. The mutated leptin receptor carried by db/db mice gave rise to an obesity-induced type 2 diabetes phenotype. Mice were bred and maintained in the animal facilities of the Vall d’Hebrón Research Institute (VHIR). With the aim of minimizing variability, mice were randomly distributed (block randomization) into groups of two mice per cage in Tecniplast GM-500 cages (36 cm × 19 cm × 13.5 cm) under standard laboratory conditions at 22 ± 2°C, with relative humidity of 50–60% and cycles of 12 h light/darkness. The cages were equipped with nesting material, absorbent bedding (BioFresh Performance Bedding 1/800 Pelleted Cellulose, Absorption Corp, Ferndale, WA, USA), ad libitum food (ENVIGO Global Diet Complete Feed for Rodents, Mucedola, Milan, Italy), and filtered water. Glycemia was measured through tail-vein blood sampling and detection with a blood glucose meter (71371-80, FreeStyle Optium Neo; Abbott). At the experimental endpoint, mice were euthanized by gradual-fill CO^2^ inhalation (20–30% chamber volume per minute) to minimize distress, followed by cervical dislocation to ensure death. All procedures were approved by the Animal Care and Use Committee of VHIR (CEEA 14/21) and complied with institutional ethical standards, the European Community directive (86/609/CEE), and the ARVO Statement for the Use of Animals in Ophthalmic and Vision Research.

#### 2.1.1 Expansion

**Figure 1:**
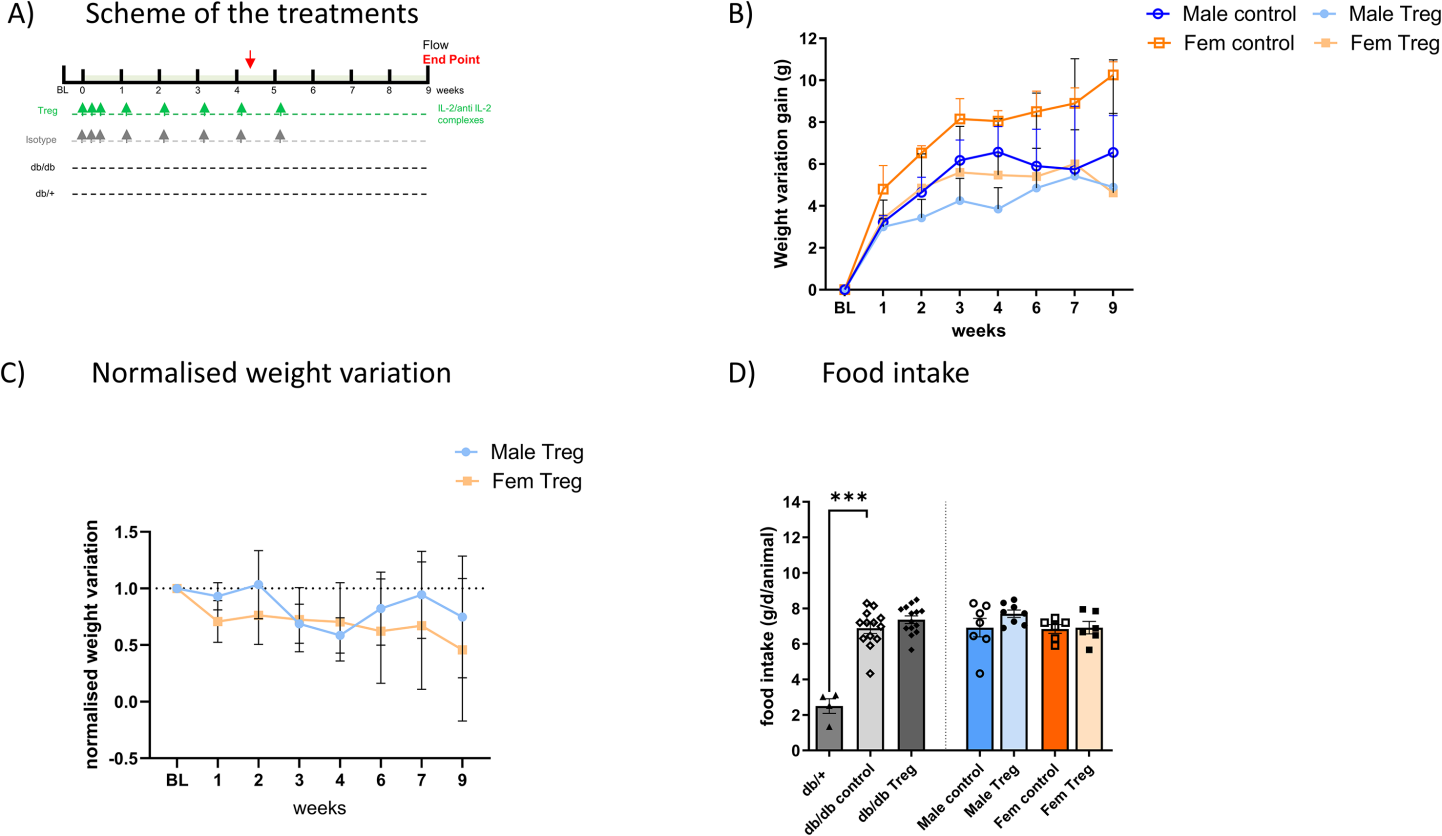
Treg Expansion induces weight loss in db/db T2D animals. **(A)** Schematic representation of the Treg expansion protocol. **(B)** Weekly weight changes in male and female animals throughout the experiment, comparing control and Treg-expanded groups. Weight variation is shown as the difference from each animal’s baseline weight at different time points. **(C)** Reduced weight-gain in Treg-expanded animals, compared between males and females, with results expressed as normalized weight variation relative to their respective control groups. **(D)** Food intake measured over six days at the experiment’s midpoint (week 4) in male and female animals, comparing control and Treg-expanded groups. Food intake is expressed as grams per day per animal (g/d/animal). n=4-8. Data expressed as mean ± s.e.m. 1-way ANOVA followed by Šídák’s multiple comparisons test. ***p<0.005.

The expansion of endogenous Treg was performed as previously described (11, 16) through the injection of IL-2/anti-IL-2 mAb (JES6-1) complexes. Briefly, recombinant mouse IL-2 (PeproTech, 1µg per animal; Cat. No. 212-12) was mixed with either anti–IL-2 monoclonal antibody (mAb) (clone JES6-1; BE0043; Abyntek; 5 µg per animal, referred as Treg group) or its isotype control (rat IgG2a, referred to as “isotype” group from here on), and incubated at 37°C for 30 min. 9-week-old mice were injected intraperitoneally (i.p.) in a final volume of 200 µL. Mice received a daily injection for 3 consecutive days on the first week and then a reminder injection once weekly for 5 weeks (Summarized in **Figure 1A**). An additional group of db/db animals non-injected was added to the study to ascertain possible alterations driven by the isotype treatment in comparison to basal db/db mice. Animals were 18–19 weeks at the end point of the experiment, representing an established but not yet advanced stage of diabetes. Male and female db/+ (non-diabetic controls), db/db (diabetic controls), and Treg-expanded db/db mice were used in this study. Unless otherwise stated, each experimental group included 4–5 animals (n = 4–5 per group). Thus, five experimental groups were analyzed in total: db/+ male/females, db/db males, db/db females, Treg-expanded db/db males, and Treg-expanded db/db females. Mice were randomly assigned to experimental groups.

### 2.2 Immunoassays

#### 2.2.1 Insulin quantification

Plasma insulin levels were determined using an ultrasensitive mouse enzyme-linked immunosorbent assay (10-1251-01, Mercodia Inc., Winston-Salem, NC, USA).

#### 2.2.2 Magnetic bead-based immunoassay

Blood obtained from the animals at the time of perfusion was collected in EDTA-tubes to avoid blood coagulation. Cytokines, C-Reactive Protein (CRP) and adiponectin concentrations were determined using the ProcartaPlex Mouse and Rat Mix & Match kit (Thermo Fisher Scientific, PPX-10-MXT2CFM and PPX-02-MXWNCYKF) following the manufacturer’s instructions. Samples were processed in a 96-well flat-bottom plate and analyzed with a Luminex MAGPIX instrument (Luminex Corporation, Austin, TX, USA) utilizing xPONENT 4.2 software (Luminex Corporation). The MAGPIX system employs a sandwich ELISA methodology, where the capture antibody is attached to fluorescently dyed magnetic microspheres, facilitating bead-based quantitative protein detection. The protein concentration was derived from standard curves through five-parameter logistic (5 PL) curve fitting conducted by the software.

#### 2.2.3 Western blot

Frozen liver samples were homogenized by sonication in 100 µL of RIPA buffer supplemented with protease inhibitor cocktail (Sigma, Cat. Nos. 04693159001 and R0278) and PhosSTOP™ (Roche, Cat. No. 04906837001). Protein concentration was determined using the Bradford Assay (Thermo Fisher Scientific, Cat. No. 23246). For Western blotting, 40 µg of protein was resolved as previously described (17), using nitrocellulose membranes. Blots were probed with antibodies against FoxP3 (Abcam, ab215602) and GAPDH (Abcam, ab8245), followed by HRP-conjugated goat anti-rabbit IgG or rabbit anti-mouse IgG. Detection was performed with WesternBright ECL HRP substrate (Advansta, K-12045-D50CA) and visualized using the LI-COR Odyssey FC 2800 Imaging System (LICORbio).

### 2.3 RT-PCR

Total RNA was extracted from mouse liver using TRIzol™ reagent (Invitrogen SA). The RNA concentration and integrity were assessed using a NanoDrop spectrophotometer (ThermoFisher Scientific). A minimum of 400 ng of RNA was reverse-transcribed into complementary DNA using the High-Capacity cDNA Reverse Transcription Kit (ThermoFisher Scientific) and Oligo(dT)18 Primer (ThermoFisher Scientific) during a predeterminate 2h thermal cycling conditions: annealing step (25°C for 10 min), polymerization step (37°C for 120 min), enzyme deactivation step (85°C for 5 min), and hold at 4°C.

Quantitative PCR (qPCR) was performed in 384-well plates (Thermo Fisher Scientific) using SYBR™ Green PCR Master Mix (Applied Biosystems, USA) and gene-specific primers for *F4/80, Il10, Il13, Pparγ*, and *B2m*. The following sequences (forward, reverse; 5′→3′) were used: *B2m* (ACTGATACATACGCCTGCAGAGTT, TCACATGTCTCGATCCCAGTAGA), *F4/80* (CTTTGGCTATGGGCTTCCAGTC, GCAAGGAGGACAGAGTTTATCGTG), *Pparγ* (TCTTAACTGCCGGATCCACAA, GCCCAAACCTGATGGCATT), *Ifnγ* (AGCAACAGCAAGGCGAAAA, CTGGACCTGTGGGTTGTTGA), *Il1b* (CTGGTGTGTGACGTTCCCATTA, CCGACAGCACGAGGCTTT), *Il6* (TCGTGGAAATGAGAAAAGAGTTG, AGTGCATCATCGTTGTTCATACA), *Il10* (CGGGAAGACAATAACTGCACCC, CGGTTAGCAGTATGTTGTCCAGC), and *Il13* (CCATACCATGCTGCCGTTGCA, TGGCTCTTGCTTGCCTTGGTGG). Reactions were run on a QuantStudio™ 7 Pro Real-Time PCR System (Applied Biosystems) with the following cycling conditions: 50°C for 2 min, 95°C for 10 min, followed by 40 cycles of 95°C for 15 s and 60°C for 1 min. A melt-curve analysis (95°C for 15 s, 60°C for 15 s, and 95°C for 15 s) was performed to confirm amplification specificity. All reactions were run in duplicate or triplicate. Relative gene expression was calculated using the 2^−ΔΔCt method, with β2-microglobulin (B2m) as the reference gene.

### 2.4 Triglycerides quantification

Triglyceride levels in mouse liver were determined using the Abcam Triglyceride Assay Kit (ab65336) with minor adaptations for tissue homogenates. Liver tissue (~100 mg), stored at −80°C, was thawed on ice, washed in PBS, and homogenized in 1 mL of 5% NP-40. Homogenates underwent two heating–cooling cycles (80–100°C, 2–5 min) to solubilize triglycerides, followed by centrifugation (max speed, 2 min). Supernatants were diluted 1:10 in water, and 50 µL per well (in duplicate) was used for quantification.

### 2.5 Statistical analysis

Statistical analysis was performed using Graph Pad Prism (GraphPad Software, Inc. version 10). Normal distribution was assessed using Kolmogorov-Smirnov tests. For comparisons involving more than two groups, 1-way ANOVA analysis was performed followed by Šídák’s multiple comparisons test. For all statistical tests, differences were considered significant with p values below 0.05.

## 3 Results

### 3.1 Treg expansion reduces weight gain in db/db mice

Animals were treated as described in **Figure 1A**, and successful Treg expansion was confirmed in the spleen and peripheral blood (11). No sex differences were observed in Treg levels or expansion efficiency (data not shown). Longitudinal weight monitoring revealed that Treg-expanded animals exhibited significantly lower weight gain compared to non-expanded control animals (**Figures 1B, C**), in both male and female db/db mice, throughout the entire duration of the experiment.

To assess whether food intake contributed to these differences, we measured daily food consumption at weeks 4 and 6. As shown in **Figure 1D**, the characteristic hyperphagia observed in db/db mice remained unaltered following Treg expansion, indicating that reduced weight gain was independent of changes in food intake.

### 3.2 Female mice exhibit improved insulin resistance following Treg expansion

**Figure 2:**
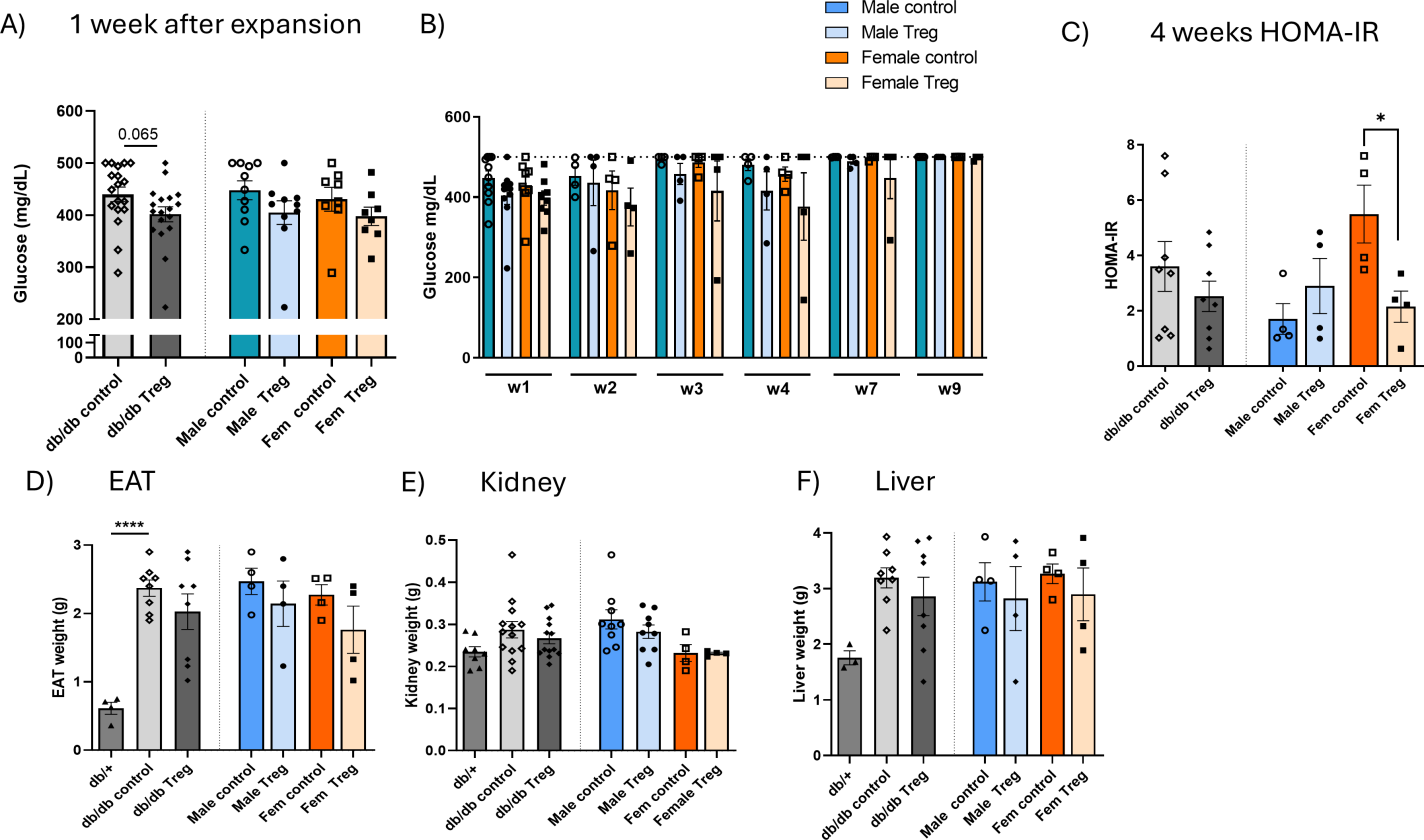
Metabolic changes associated with Treg expansion. **(A)** Blood glucose levels measured one week after endogenous Treg expansion in Treg-expanded and control db/db animals, presented separately by sex. n=8-18. **(B)** Longitudinal evolution of blood glucose levels over the 9-week experimental period in male and female db/db mice. **(C)** Homeostatic Model Assessment for Insulin Resistance (HOMA-IR), calculated using [HOMA-IR=[Glucose (mg/dL)×Insulin (µU/mL)]/405, measured at the midpoint of the experiment (weeks 4–5) and at the endpoint, in control and Treg-expanded db/db animals, stratified by sex. **(D–F)** Tissue weights at the endpoint, including epididymal adipose tissue (EAT) **(D)**, kidney **(E)** n=3-4, and liver **(F)** n=4-8, in T2D db/db and db/+ mice, comparing Treg-expanded and control groups, presented separately by sex. Data expressed as mean ± s.e.m. 1-way ANOVA followed by Šídák’s multiple comparisons test. *P < 0.05; ****P < 0.001.

Blood glucose levels were monitored throughout the experiment. One-week post-expansion, a non-significant trend toward lower glucose levels was observed in Treg-expanded animals (**Figure 2A**). Over the following weeks, blood glucose levels remained elevated in both Treg-expanded and control groups; however, female db/db mice exhibited a slight tendency toward lower glucose levels compared to their male counterparts (**Figure 2B**).

To further investigate insulin sensitivity, HOMA-IR was calculated at the experiment’s midpoint (week 4) and at the endpoint (*data not shown*). At week 4, a significant reduction in HOMA-IR was observed in female Treg-expanded mice but not in males (**Figure 2C**). At the study endpoint, HOMA-IR values remained unchanged between groups, likely due to the marked hyperglycemia present at this stage, when glucose levels frequently exceeded 500 mg/dL and accurate assessment became unreliable.

### 3.3 Tissue weight analysis in Treg-expanded mice

To assess potential metabolic alterations, we measured the weight of tissues commonly affected in T2D, including epididymal adipose tissue (EAT) (**Figure 2D**), kidney (left) (**Figure 2E**), and liver (**Figure 2F**). Although differences were not statistically significant, female Treg-expanded db/db mice consistently exhibited lower tissue weights, most notably in the liver, suggesting a potential beneficial of Treg expansion on metabolic tissue remodeling.

### 3.4 Impact of Treg expansion on systemic inflammation

**Figure 3:**
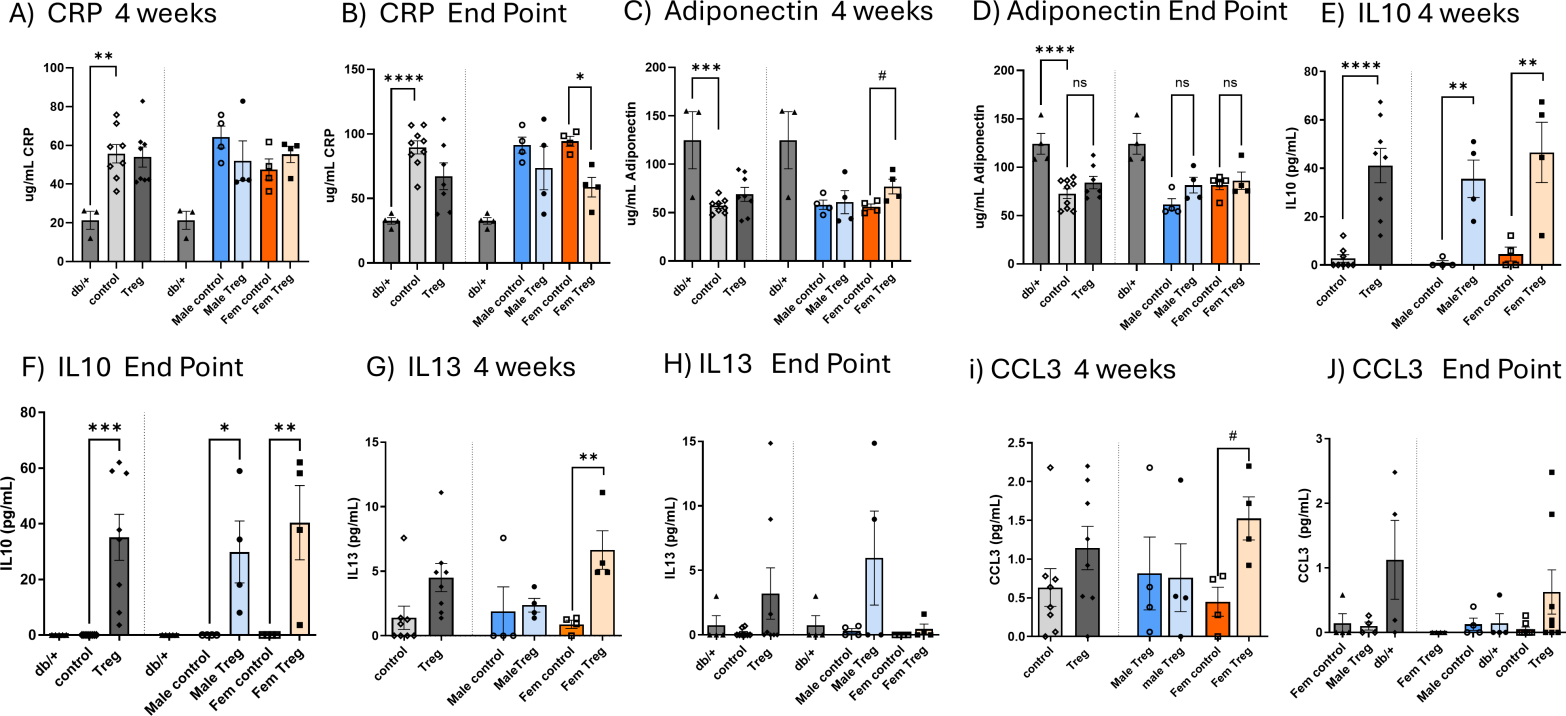
Impact of Treg expansion on CRP and adiponectin levels and circulating levels of IL-10, IL-13, and CCL3. Circulating plasma concentrations of CRP **(A, B)** and adiponectin **(C, D)** measured at the midpoint of treatment [4 weeks; **(A**, **C)**] and at the study endpoint **(B, D)** in db/db diabetic mice receiving either control or Treg expansion treatment, as well as in non-diabetic db/+ mice. Data are further stratified by sex. Plasma concentrations of anti-inflammatory cytokines IL-10 **(E, F)** and IL-13 **(G, H)**, and the chemokine CCL3 **(I, J)**, were measured in diabetic mice subjected to Treg expansion, control diabetic mice, and non-diabetic db/+ mice. In each graph, data are first shown for both sexes combined, followed by separation by sex. Graphs **(E, G, I)** show cytokine levels at 4 weeks; graphs **(F, H, J)** show levels at the study endpoint. Values are presented as mean ± s.e.m. n=3-4. Statistical analysis was performed using one-way ANOVA followed by Šídák’s multiple comparisons test. *P < 0.05; **P < 0.01; ***P < 0.005; ****P < 0.001.

We next investigated the impact of Treg expansion on CRP and adiponectin in plasma. CRP levels were elevated at both time points studied: at 4 weeks (mid-treatment, **Figure 3A**) and at the endpoint (**Figure 3B**) in db/db mice. Interestingly, at the endpoint, only female mice that underwent Treg expansion exhibited a significant decrease in CRP levels compared to their respective controls (**Figure 3B**).

Conversely, the anti-inflammatory marker adiponectin was reduced in T2D db/db animals at both 4 weeks and the endpoint (**Figures 3C, D**, respectively). Notably, at 4 weeks, female mice that underwent Treg expansion displayed a significant increase in plasma adiponectin levels (**Figure 3C**), suggesting a potential sex-dependent effect of Treg expansion on anti-inflammatory pathways in T2D.

To further explore the effects of Treg expansion, we assessed changes in plasma levels of key pro- and anti-inflammatory cytokines. While no significant differences were observed for the pro-inflammatory cytokines IL-1β, IL-6, TNFα, and CCL2 (data not shown), we did detect significant alterations in other cytokines (**Figures 3E-J**).

As shown in **Figures 3E**, **F**, Treg-expanded db/db mice exhibited a significant increase in plasma of the anti-inflammatory cytokine IL-10, at both 4 weeks (mid-experiment) and at the endpoint (**Figures 3E, F**, respectively). This increase was observed in both males and females though females demonstrated a more pronounced effect at the endpoint.

Additionally, the also anti-inflammatory IL-13 and the chemokine CCL3 were significantly increased at 4 weeks in female T2D db/db mice subjected to Treg expansion (**Figures 3G, I**). Although these increases were no longer statistically significant at the endpoint (**Figures 3H, J**), the data showed a sustained, though attenuated, modulation of these cytokines over time. Together, these findings indicate that Treg expansion exerts a transient but biologically meaningful, sex-dependent enhancement of anti-inflammatory and chemokine responses in T2D.

### 3.5 Impact of Treg expansion on the db/db liver

**Figure 4:**
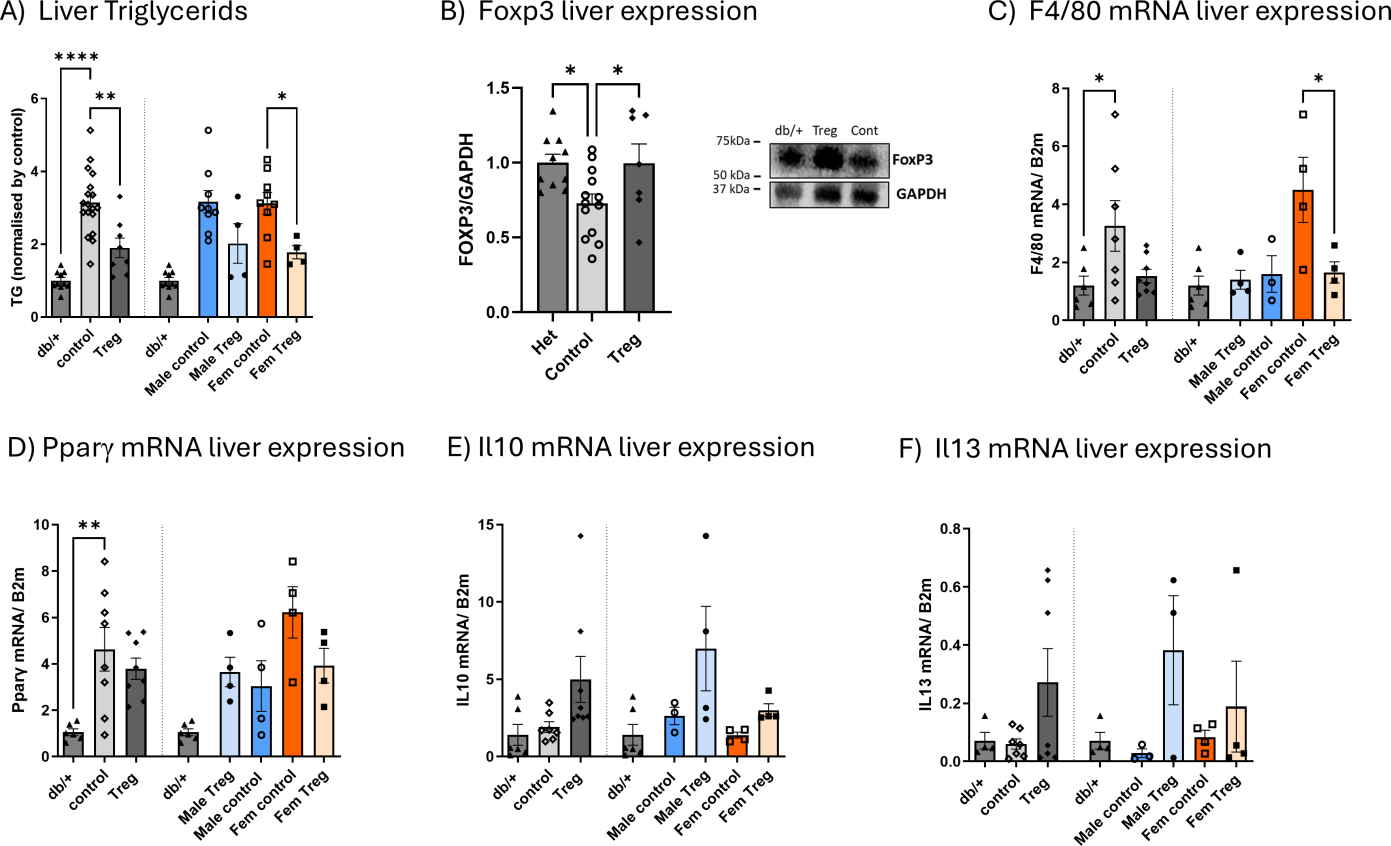
Impact of Treg expansion on hepatic steatosis, Foxp3 expression, and inflammatory markers. Hepatic triglyceride (TG) content **(A)** measured at the study endpoint in db/db diabetic mice receiving either control or Treg expansion treatment, as well as in non-diabetic db/+ mice, expressed relative to the mean value of non-diabetic controls. Hepatic Foxp3 expression **(B)** was assessed by Western blot, with representative blots shown. Expression of *F4/80* **(C)**, *Pparγ* **(D)**, *Il10* **(E)**, and *Il13* **(F)** was quantified by RT–PCR in the same groups at the study endpoint. Data are stratified by sex, with each graph showing values for both sexes combined, followed by separation by sex. Values are presented as mean ± s.e.m.; n = 3–4 per group. Statistical analysis was performed using one-way ANOVA followed by Šídák’s multiple comparisons test. *P < 0.05; **P < 0.01; ****P < 0.001.

Considering the pronounced trend toward decreased liver weight in Treg-expanded db/db mice, we next evaluated hepatic metabolic and inflammatory changes. Triglyceride (TG) content was significantly reduced following Treg expansion, with the effect more pronounced in females (**Figure 4A**). Hepatic expression of Foxp3, a key transcription factor for Treg development and function, was decreased in db/db mice but restored to non-diabetic levels by Treg expansion, consistent with our spleen and blood findings (11) (**Figure 4B**). To further characterize hepatic inflammation, we assessed the expression of key cytokines by RT–PCR. No significant changes were observed in *Ifnγ*, *Il1β*, or *Il6* (*data not shown*). However, *F4/80* expression was reduced in Treg-expanded diabetic mice, particularly in females (**Figure 4C**). *Pparγ*, which was elevated in diabetic animals, showed a mild trend toward reduction after Treg expansion, again more evident in females (**Figure 4D**). Notably, the anti-inflammatory cytokines *Il10* and *Il13* displayed a clear trend toward increased expression in Treg-expanded animals (**Figures 4E, F**, respectively).

## 4 Discussion

Immune system dysregulation, involving both the innate and adaptive immune responses, is a key pathogenic factor in T2D (3–7). In general, adult females exhibit stronger innate and adaptive immune responses than males. This is characterized by increased macrophage and neutrophil activation, as well as enhanced phagocytic capacity. Regarding the adaptive immune system, females demonstrate higher T cell activation and proliferation, increased cytotoxic T cell activity, and an elevated number of B cells. Additionally, some studies suggest lower numbers of Tregs in females compared to males (18), further underscoring sex-specific immune differences that may contribute to T2D pathogenesis.

In our study, Treg expansion did not induce significant metabolic changes in male mice, apart from a reduction in weight gain. However, under the same Treg expansion protocol, female mice exhibited clear metabolic changes. Notably, weight gain was significantly less pronounced in females. This effect was not attributable to changes in food intake. Furthermore, mid-experiment assessments revealed a significant reduction in HOMA-IR, thus suggesting a reduction in insulin resistance.

Despite this metabolic improvement, liver, epididymal fat, and kidney weights were not significantly reduced in Treg-expanded female mice but showed a modest downward trend. Further studies, including additional functional metabolic assessments and detailed histological or molecular analyses of these organs, will be required to elucidate the meaning of these trends. Interestingly, CRP—a well-established marker of systemic inflammation and a predictor of cardiovascular risk in T2D patients—was significantly decreased at the end of the study in female mice. This suggests an attenuation of chronic inflammation, a key feature of T2D and a meaningful factor for developing cardiovascular disease.

Additionally, adiponectin, an anti-inflammatory adipokine known to enhance insulin sensitivity and regulate glucose metabolism, was elevated in the middle of the treatment in Treg-expanded female T2D mice. This increase in adiponectin, along with the observed CRP reduction, reflects a modulation of the systemic inflammatory profile, further supporting the beneficial impact of Treg expansion on metabolic health. Changes in female db/db mice peripheral blood cytokine levels further corroborated this immune modulation. IL-10 and IL-13 are known to suppress pro-inflammatory cytokine production and play a role in insulin resistance, respectively (19, 20).

Treg expansion in db/db mice not only reduced liver weight and triglyceride content but also restored hepatic Foxp3 expression, indicating enhanced local Treg presence. These effects were associated with a decreased expression of the *macrophage marker F4/80* and a trend towards a lower *Pparγ* and higher anti-inflammatory cytokines (*Il10*, *Il13*), paralleling systemic increases in IL-10, IL-13, and CCL3 observed earlier in the experiment. The concordance between hepatic and systemic anti-inflammatory shifts suggests that Treg expansion attenuates liver inflammation both directly, via local immune modulation, and indirectly, through broader immunoregulatory effects. Notably, these benefits were generally more pronounced in females, highlighting a potential sex-dependent responsiveness to Treg-based interventions in T2D.

Sex-related immune differences in metabolism have been well documented in the literature. In young females, estrogen suppresses visceral adipose tissue (VAT) inflammation, reducing the need for Treg recruitment (21). In contrast, males display increased VAT inflammation and a higher number of Tregs with a distinct phenotype. This is driven by a male-specific circuit where androgens promote inflammation and support the expansion of IL-33–producing stromal cells. IL-33 and pro-inflammatory cytokines act together to recruit Tregs into male VAT. These Tregs adopt a transcriptional program influenced by BLIMP1, which contributes to maintaining metabolic balance; disruption of this pathway impairs glucose regulation. With aging, however, females undergo a detrimental shift—characterized by increased pro-inflammatory T cells and reduced anti-inflammatory Tregs in adipose tissue (22). Our findings highlight a sex-specific response to Treg expansion in T2D, with females demonstrating more pronounced metabolic and inflammatory modulation. This effect may be mediated by IL-13, adiponectin, and a reduction in systemic inflammation.

Tregs play a crucial role in suppressing inflammation, partly through their ability to secrete IL-13, which stimulates IL-10 production in macrophages (19, 20, 23). Additionally, Tregs themselves produce and secrete IL-10, reinforcing their anti-inflammatory function (23). Another key factor is CCL3, a chemokine produced by Tregs (24), which acts as a chemoattractant, recruiting Tregs to sites of injury (24). CCL3 secretion by Tregs also facilitates the recruitment of CD4+ and CD8+ T cells (24), further underscoring the complexity of Treg-mediated immune regulation in T2D.

Given the immunological differences between sexes, it is plausible that Treg-based therapy confers greater metabolic benefits in female T2D mice than in males. This may reflect intrinsic sex-based immune disparities, including heightened immune responsiveness in females or reduced functional capacity of male-derived Tregs to mitigate adipose inflammation. It is also possible that the therapeutic regimen was insufficient to sustain Treg expansion in males at levels required to counter the more pro-inflammatory milieu of male adipose tissue (21). A limitation of this study is the lack of direct functional assessment of Treg suppressive capacity. Indeed, an impairment of Treg suppressive function has been documented in diabetes (10), which can differ between sexes, but these differences are also dependent on type of diabetes and the stage of the disease (25, 26). Therefore, functional differences in the expanded Treg between sexes cannot be excluded. Nevertheless, the primary focus of our study was to evaluate systemic metabolic and inflammatory outcomes following Treg expansion in T2D, and the conclusions are based on these robust functional readouts.

Overall, this study contributes to the growing body of knowledge regarding sex-based differences in immune responses, highlighting the ongoing need for deeper investigation into immune-related diseases from a sex-specific perspective. Further research is warranted to explore the functional disparities between male and female Tregs in diabetic conditions. Additionally, evaluating the impact of a more prolonged or sustained Treg expansion protocol may yield greater metabolic improvements and provide deeper insights into the therapeutic potential of Treg-based interventions in T2D.

## Data Availability

The raw data supporting the conclusions of this article will be made available by the authors, upon reasonable request, without undue reservation.
